# Segregation of lipids near acetylcholine-receptor channels imaged by cryo-EM

**DOI:** 10.1107/S2052252517005243

**Published:** 2017-05-02

**Authors:** Nigel Unwin

**Affiliations:** a MRC Laboratory of Molecular Biology, Francis Crick Avenue, Cambridge Biomedical Campus, Cambridge CB2 0QH, England

**Keywords:** nicotinic acetylcholine receptor, cholesterol, lipid microdomain, cryo-EM, helical image reconstruction

## Abstract

Rapid communication at the chemical synapse depends on the action of ion channels residing in the postsynaptic membrane. The channels open transiently upon the binding of a neurotransmitter released from the presynaptic nerve terminal, eliciting an electrical response. Membrane lipids also play a vital but poorly understood role in this process of synaptic transmission. The present study examines the lipid distribution around nicotinic acetylcholine (ACh) receptors in tubular vesicles made from postsynaptic membranes of the *Torpedo* ray, taking advantage of the recent advances in cryo-EM. A segregated distribution of lipid molecules is found in the outer leaflet of the bilayer. Apparent cholesterol-rich patches are located in specific annular regions next to the transmembrane helices and also in a more extended ‘microdomain’ between the apposed δ subunits of neighbouring receptors. The particular lipid distribution can be interpreted straightforwardly in relation to the gating movements revealed by an earlier time-resolved cryo-EM study, in which the membranes were exposed briefly to ACh. The results suggest that in addition to stabilizing the protein, cholesterol may play a mechanical role by conferring local rigidity to the membrane so that there is productive coupling between the extracellular and membrane domains, leading to opening of the channel.

## Abstract 2

A single-particle helical reconstruction from cryo-EM images reveals a segregated distribution of lipids around acetylcholine-receptor channels in the outer leaflet of the bilayer. The existence of a cholesterol-rich microdomain next to the receptor suggests how this lipid ensures a productive conformational change when acetylcholine binds, triggering the channel to open.

## 1. Introduction   

Rapid communication in the nervous system takes place by synaptic transmission, a process in which a neurotransmitter released from a nerve terminal binds transiently to ion channels located in the oppositely facing postsynaptic membrane of the target cell, stimulating the channels to open and effecting a change in membrane potential. Models for the conformational change underlying the postsynaptic response have been derived from both X-ray and cryo-EM studies of several kinds of transmitter-gated ion channel, using recombinant detergent-solubilized protein [for channels in the acetylcholine (ACh) receptor family, see Hibbs & Gouaux, 2011 ▸; Miller & Aricescu, 2014 ▸; Hassaine *et al.*, 2014 ▸; Du *et al.*, 2015 ▸; Morales-Perez *et al.*, 2016]. These studies have provided a wealth of structural insight at near-atomic resolution on the channels themselves. However, membrane lipids also play a vital role (daCosta & Baenziger, 2013 ▸), and the actual physiological mechanism is therefore best understood by analyzing both the channel and the lipid components together in their natural membrane setting.

Postsynaptic membranes isolated from the *Torpedo* electric organ provide such a setting. They form tubular vesicles having the same lipid composition as, and near-equivalent architecture to, the postsynaptic membrane *in situ* (Heuser & Salpeter, 1979 ▸; Unwin, 2013 ▸). Furthermore, the tubes are amenable to crystallographic analysis, since their constituent ion channels, ACh receptors, arrange on a helical surface lattice. As a result, it has been possible to obtain an atomic model of the membrane-bound ACh receptor, giving molecular details of the five subunits (α_δ_, α_γ_, β, γ and δ) encircling the central ion path (Miyazawa *et al.*, 2003 ▸; Unwin, 2005 ▸). Most recently, a time-resolved cryo-EM study of ACh-reacted tubes allowed a simple mechanical description of how ACh opens the channel (Unwin & Fujiyoshi, 2012 ▸). In brief, ACh binding to α_δ_ and α_γ_ triggers small concerted displacements in the extracellular domain, and these communicate to the membrane domain through the action of α_γ_ pushing against the β subunit, which tilts outwards in the membrane to open the pore. Although other changes, such as straightening of the adjacent α_γ_ and δ pore-lining helices, also occur, the displace­ment outwards of β near the outer membrane surface is the motion that most directly affects the lipids.

Here, we investigate the likely role played by the lipids in facilitating this transient conformational change, taking advantage of the recent advances in direct-electron detector technology and computer software, which enable improved definition of low-contrast features in the image. We find that the outer leaflet of the bilayer has a segregated distribution of lipids, indicated by patches of differing density in the region of the phospholipid headgroups. The low-density patches, which we identify with cholesterol-rich areas, are at equivalent annular sites (involving helices M1 and M4) around the receptor and in a more extended space between the apposed δ subunits of neighbouring receptors. This distribution suggests that cholesterol may play a dual role in (i) stabilizing the transmembrane α-helical organization and (ii) conferring local rigidity to the membrane so that there is productive coupling between the extracellular and membrane domains, leading to opening of the channel. Similar facilitating roles for the lipids have been proposed for other membrane proteins that undergo large-scale conformational changes (see, for example, Cornelius *et al.*, 2015 ▸; Landreh *et al.*, 2016 ▸).

## 2. Methods   

### 2.1. Specimen preparation   

Tubular postsynaptic membrane vesicles were prepared from fresh *Torpedo* electric organ as described by Kubalek *et al.* (1987 ▸) and suspended in 100 m*M* sodium cacodylate, 1 m*M* calcium chloride pH 7.0. Aliquots (3.8 µl) of this solution were applied to holey carbon support grids and blotted to retain the specimens in a thin aqueous film before plunging into liquid nitrogen-cooled ethane. Possible heterogeneity in lipid composition owing to individual or seasonal variation was minimized in these experiments by using tubes from only one fish.

### 2.2. Cryo-EM data collection and image processing   

We chose ∼750 Å diameter tubes belonging to the (−17, 5) helical family (Miyazawa *et al.*, 1999 ▸) for the structure analysis, which applies the single-particle method of helical image reconstruction (Egelman, 2000 ▸; Sachse *et al.*, 2007 ▸; He & Scheres, 2017 ▸) rather than the Fourier–Bessel method (Toyoshima & Unwin, 1990 ▸; Beroukhim & Unwin, 1997 ▸) applied in earlier studies. Both methods correct for long-range variations in the surface lattice and for distortions, such as bending and out-of-plane tilt, which are inevitably present in ACh-receptor tubes because of their shell-like architecture and the fluid nature of the lipid matrix in which the protein is embedded. The (−17, 5) tubes have dihedral (*D*1) symmetry and form a single-start helix with average twist and rise values of 147.0° and 5.9 Å, respectively.

**Figure 1:**
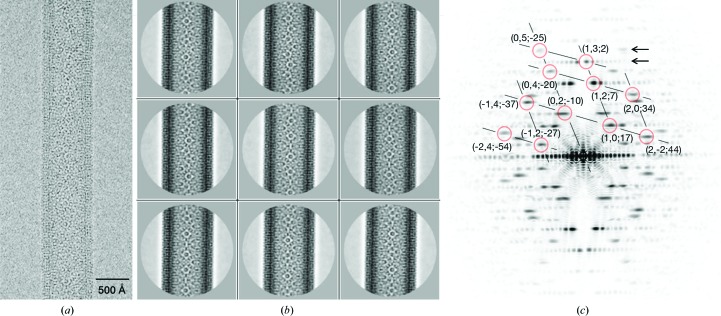
Two-dimensional classification and image selection. (*a*) Micrograph of a (−17, 5) tube recorded at 2.4 µm underfocus with a Falcon 2 direct-electron detector. (*b*) Examples of good class averages. (*c*) Fourier transform of a good class average, showing ‘pairing layer lines’ (arrows) reflecting the dimeric arrangement of receptors on the tube surface lattice (Brisson & Unwin, 1984 ▸). The figures in parentheses denote the (*h*, *k*; *n*) indices for major low-resolution layer lines (Toyoshima & Unwin, 1990 ▸). Data from class averages where the pairing layer lines [(0, 5; −25) and (1, 3; 2)] were weak or non-existent were omitted from the subsequent image-processing steps.

The specimens were imaged with an FEI Titan Krios transmission electron microscope incorporating a 70 µm diameter objective aperture and operating in nanoprobe mode at an accelerating voltage of 300 kV. Micrographs were recorded on a Falcon 2 4096 × 4096 pixel direct-electron detector after searching for straight (−17, 5) tubes spanning holes in the carbon support film (Fig. 1 ▸ *a*). The calibrated pixel size was 1.34 Å and the total dose on the specimen was 35 e Å^−2^ fractionated over 22 frames.

We used 295 micrographs of the tubes, recorded with an underfocus range of 1–3 µm and selected by inspection of their Fourier transforms. Each tube image was divided into overlapping segments using a box size of 1024 × 1024 pixels and an inter-box spacing of 100 pixels. Micrograph frame stacks were drift-corrected using *UCSF MotionCorr* (Li *et al.*, 2013 ▸), and contrast-transfer function parameters were determined locally along the tube axis using *Gctf* (Zhang, 2016 ▸). For three-dimensional classification, an initial 20 Å resolution model was generated by Fourier–Bessel synthesis from structure-factor terms along the layer lines (Miyazawa *et al.*, 1999 ▸). All subsequent image-processing steps were performed in *RELION* (Scheres, 2012 ▸; He & Scheres, 2017 ▸) after binning the images times two.

Reference-free two-dimensional classification was applied to the extracted segments, yielding 34 class averages (Fig. 1 ▸ *b*), of which 80% were considered good on the basis of the quality of their Fourier transforms (Fig. 1 ▸ *c*). Rejected segments included disordered or defective regions of tubes and regions where the projected *p*2 symmetry (indicated the presence of ‘pairing layer lines’; arrows in Fig. 1 ▸ *c*) was weak or absent. Only segments associated with the good classes were retained for subsequent three-dimensional classification.

Three-dimensional classification was performed on 7196 extracted segments using a cylindrical mask of inner and outer radii sufficient to include all densities comprising the tube with a margin of ∼40 Å on either side. The ‘central *Z*-length’, used for searching and applying helical symmetry in real space, was 10% of the box size. In an initial step, three class averages were generated. One of these class averages (class 1; 2563 segments) showed better alignment errors and resolution than the other two. Moreover, the reconstructed tube from this class had the expected diameter, whereas the reconstructed tubes from the other two classes, while displaying the same features, were slightly wider. Based on these criteria, the class 1 segments appeared to be the least affected by flattening owing to insufficient thickness of the ice and/or other possible sources of heterogeneity, such as variation in lipid content. This class was therefore refined subsequently, yielding a resolution for the final three-dimensional density map of 8.4 Å (FSC = 0.143 threshold, two independently refined half-data sets).

**Figure 2:**
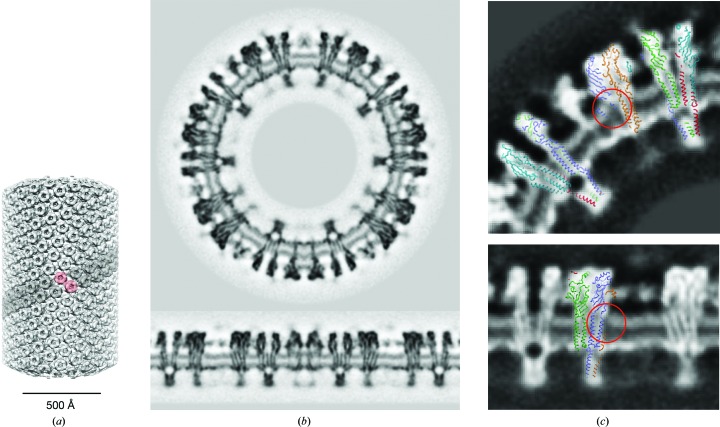
Single-particle helical reconstruction from (−17, 5) tubes. (*a*) Masked-out volume showing the helical arrangement of ACh receptors; a δ–δ linked dimer of receptors and a ribbon of dimers are shaded in pink and grey, respectively. (*b*) Central cross-section (top) and radial section (bottom) from the three-dimensional density map cutting through receptors and the lipid-bilayer matrix in which they are embedded. (*c*) Enlargements of regions in (*b*) with atomic models of the ACh receptor (PDB entry 2bg9) superimposed. Circles identify areas next to the δ subunit where densities corresponding to the outer phospholipid headgroups are weak or missing; the adjacent helices are M1 (lower circle), and M4 and M1 (upper circle). Subunit colours: α_γ_, red; α_δ_, orange; β, green; γ, cyan; δ, blue. Inverted contrast.

An atomic model of the ACh receptor (PDB entry 2bg9; Unwin, 2005 ▸) was fitted to the densities using *UCSF Chimera* (Pettersen *et al.*, 2004 ▸) and was incorporated in Fig. 2 ▸ using *PyMOL* (DeLano, 2002 ▸).

## 3. Results   

### 3.1. Architecture of a tubular vesicle   

The single-particle helical reconstruction of the (−17, 5) tubes (Fig. 2 ▸ *a*) shows very similar molecular details as established using the alternative Fourier–Bessel approach. The receptors form dimers linked by a disulfide bridge between the δ subunits of neighbouring molecules (Chang & Bock, 1977 ▸; Brisson & Unwin, 1984 ▸), as indicated by the pink shading in Fig. 2 ▸(*a*). The helical surface lattice is built from ribbons of dimers (grey shading), which associate side by side.

Cross-sectional slices through the three-dimensional map normal to the membrane plane (Fig. 2 ▸ *b*) are dominated by paired tracks of density corresponding to the phospholipid headgroups of the lipid bilayer and by irregular blocks of density corresponding to individual receptors packed tightly in the lipid matrix. The extracellular domain of the receptor, built around a β-sandwich core, extends about 60 Å from the outer membrane surface. The intracellular domain, which is shaped largely by a conical arrangement of α-helices, extends about 40 Å from the inner membrane surface. The receptor has an all-α-helical transmembrane domain, with four helices (M1, M2, M3 and M4) per subunit. The helices are sufficiently long to span the outer phospholipid headgroups as well as the central low-density hydrophobic portion of the bilayer (see §[Sec sec3.2]3.2).

To evaluate the protein structure, we fitted an atomic model of the ACh receptor (PDB entry 2bg9) to the densities (see §[Sec sec2]2). Fig. 2 ▸(*c*) shows slices through the atomic model superimposed on several views of the channel, with each subunit identified in a different colour. As can be seen, most of the protein densities are accounted for by the atomic model. An exception is the density arising from a short transverse helix, which has been modelled in the related α4β2 nicotinic and 5HT_3_ receptor structures (Hassaine *et al.*, 2014 ▸; Morales-Perez *et al.*, 2016 ▸) and lies on the inner membrane surface. The clustering protein rapsyn may be responsible for some of the density at the base of the receptor, but is otherwise invisible because it forms variable networks that do not match the helical symmetry of a tube (Zuber & Unwin, 2013 ▸).

### 3.2. The lipid bilayer   

The single-particle helical method applied in the present study, unlike the Fourier–Bessel helical method, uses classification to ensure radial uniformity of the tubes and does not rely on the averaging of equatorial Fourier terms affected by the edges of the boxed-out areas, which are prone to error. As a result, the radial density distribution is now more accurate and the membrane appears with better definition than could be achieved previously. The outer leaflet of the bilayer is of particular interest because it is the portion that is most significantly implicated in the conformational change to open the channel.

The outer leaflet presents a fairly uniform ∼10 Å thick band of density, arising from the phospholipid headgroups, that occupies almost the entire space between the embedded helices of the receptors. However, in several locations immediately adjacent to a protein surface, the densities comprising the band are diminished or missing altogether (circles in Fig. 2 ▸ *c*).

**Figure 3:**
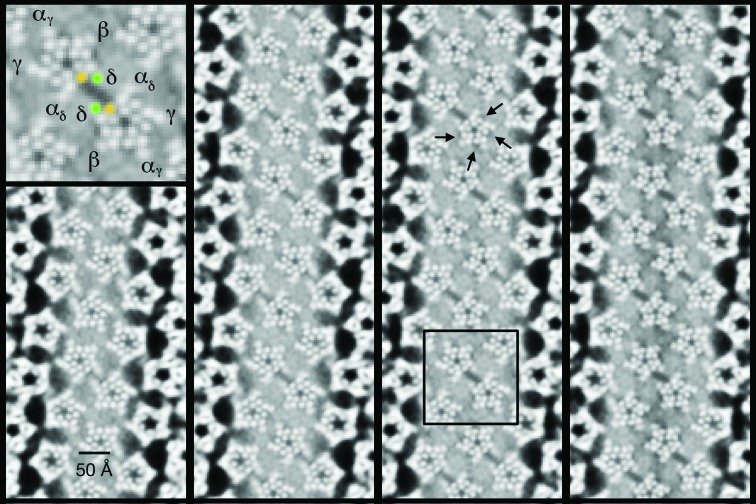
Sections tangential to the tube axis spanning the outer phospholipid headgroup region of the lipid bilayer. Low-density patches are present in the space between the δ subunits of neighbouring receptors (see, for example, the box in the third panel) and at the M1/M4 lipid–helix interface of the remaining four subunits (arrows). Inset: enlargement of the boxed dimer of receptors and the central low-density microdomain, identifying individual subunits and the M1 (orange) and M4 (green) helices of δ. The panels are at 2.7 Å intervals from the outermost side (left) to the innermost side of the headgroup region. Inverted contrast.

Sections tangential to the tube axis, encompassing the outer phospholipid headgroups, provide a more complete picture of these ‘low-density patches’ next to the protein surfaces. Fig. 3 ▸ shows four such sections extending from one side of the headgroup region to the other. The helices of the embedded receptors are now displayed in cross-section, arranged pentagonally around the water-filled pore, with densities from the lipids occupying the space surrounding them. Two kinds of low-density patch are apparent within the otherwise relatively uniform densities associated with the phospholipid headgroups. The largest occupies the area between the δ subunits of neighbouring receptors (box in Fig. 3 ▸) and is at right angles to the slices through the same region circled in Fig. 2 ▸(*c*). This patch forms a ‘microdomain’: an elongated (∼26 × 11 Å) parallelogram framed on its sides by the polar phospholipid headgroups and on its ends by the helices M1 and M4 (inset in Fig. 3 ▸). In addition, there are smaller patches located next to the M1 and M4 helices of the β, α_γ_, γ and α_δ_ subunits (arrows in Fig. 3 ▸). Both kinds of patch appear to extend across the entire headgroup region, although the details of the smaller patches are less clear.

## 4. Discussion   

Most structural studies of the roles played by lipids in the function of membrane proteins are conducted on the protein in detergent or in an artificial membrane-like environment, viewing lipid molecules that are immobilized on the protein surface. In this regard, single-particle cryo-EM of detergent-solubilized protein combined with lipid-nanodisc technology offers a promising approach (Bayburt *et al.*, 2002 ▸; Gao *et al.*, 2016 ▸). Structure determination based on reconstituted proteoliposomes (see, for example, Gonen *et al.*, 2005 ▸; Wang & Sigworth, 2009 ▸; Kudryashev *et al.*, 2016 ▸) provides a potential alternative route. However, none of these methods are likely to recapitulate precisely the lipid environment as it exists *in situ*. Here, by imaging and reconstructing the unperturbed natural membrane, we obtain the most complete physiological perspective, showing how native lipids are distributed near the protein in the outer leaflet of the native bilayer. Low-density patches are visible among the phospholipid headgroups (Fig. 3 ▸), indicating that the lipid composition is modified not only at specific annular sites around the protein but also in a more extended microdomain bordering the protein surfaces.

The resolution achieved from the limited number of images has not been sufficient to define any of the lipids creating these low-density patches as discrete molecular entities. However, we can confidently interpret the patches to reflect areas that are enriched in (or composed entirely of) cholesterol, which has a much smaller headgroup than the other lipids. For example, the microdomain within the phospholipid headgroup region (inset in Fig. 3 ▸) gives rise to a density equal to that of water in the pore at the centre of the receptor (Supplementary Fig. S1). Cholesterol in the underlying membrane accounts for this density, since it exposes only a hydroxyl and contributes no mass this far from the hydrophobic core of the membrane. Furthermore, the fraction of the outer leaflet of the bilayer occupied by the microdomain (∼6%) is readily furnished by available cholesterol molecules, which have a concentration of at least 35 mol% in *Torpedo* postsynaptic membrane (Rotstein *et al.*, 1987 ▸). Phospholipids with relatively small headgroups could not be responsible for the low density because they are present in only very small amounts (for example, phosphatidic acid at <0.5 mol%; Rotstein *et al.*, 1987 ▸).

An association of cholesterol with the ACh receptor has been well documented (for a recent review, see Barrantes, 2010 ▸), despite its absence from the structures of all ACh-receptor family members solved to date. Cholesterol plays an essential part in enabling the classical physiological transitions associated with rapid switching of the protein between closed (or resting), open and desensitized states (Criado *et al.*, 1982 ▸; Ochoa *et al.*, 1983 ▸; Sunshine & McNamee, 1992 ▸; Ryan *et al.*, 1996 ▸; Rankin *et al.*, 1997 ▸; Hamouda *et al.*, 2006 ▸). The protein adopts an uncoupled conformation, in which the pore cannot open in response to ACh binding, in reconstituted membranes lacking anionic lipids and this neutral lipid (daCosta & Baenziger, 2009 ▸). Evidently, the high concentration of cholesterol in the *Torpedo* postsynaptic membrane is needed for normal synaptic activity, since the functionality of the receptor diminishes progressively as the concentration is lowered from the natural amount (see, for example, Rankin *et al.*, 1997 ▸; Hamouda *et al.*, 2006 ▸).

**Figure 4:**
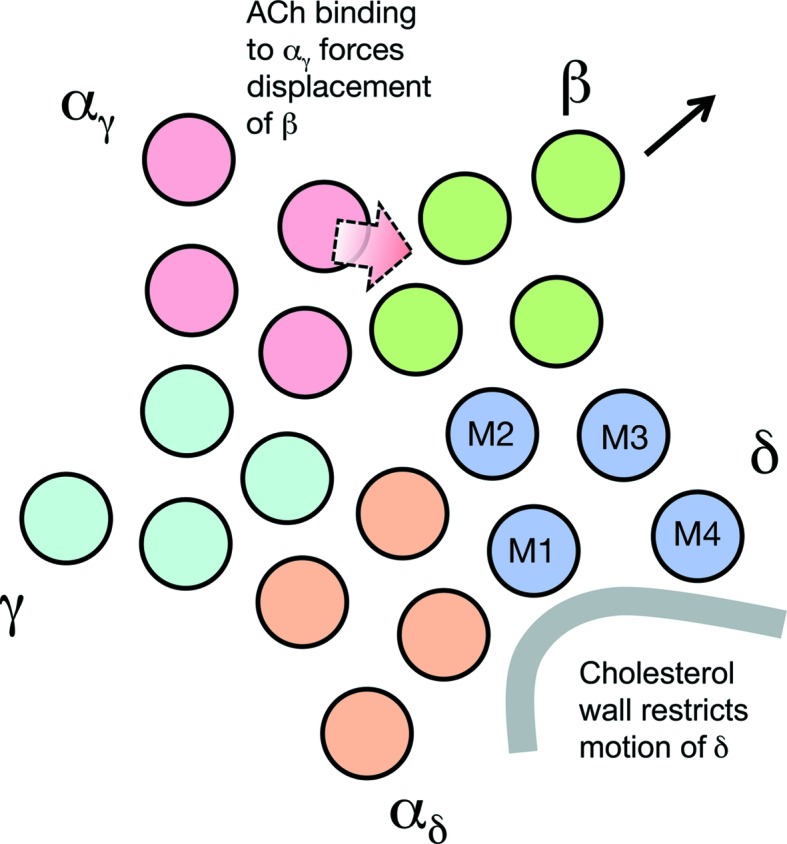
The positioning of a cholesterol-rich microdomain against the δ subunit suggests that cholesterol may play a mechanical role, restricting the mobility of δ so that β can tilt outwards to open the channel. The movements leading to channel opening, depicted in the figure, are based on the results described in Unwin & Fujiyoshi (2012 ▸). ACh binding to the receptor in the extracellular domain causes α_γ_ to push against the extracellular part of β (wide arrow), which is linked *via* (flexible) loops to the α-helical membrane-spanning part. As a result, the α-helical part of β tilts outwards (thin arrow).

How does the inferred segregation of cholesterol into areas where it is enriched (arrows and box in Fig. 3 ▸) influence the ACh-induced conformational change to open the channel? Clearly, specific annular lipid–protein interactions may be required to stabilize the α-helical organization in the membrane: for example, to restrict relative motion between the implicated helices M1 and M4 (which does not occur in any of the subunits; Unwin & Fujiyoshi, 2012 ▸). Indeed, molecular-dynamics simulations predict that cholesterol binds in the groove between M1 and M4 (Brannigan *et al.*, 2008 ▸). However, the presence of a microdomain next to the δ subunit, which is large enough to incorporate several cholesterol molecules (Supplementary Fig. S2), hints that a less direct, physical involvement may also be important (Fig. 4 ▸).

As sketched in Fig. 4 ▸, the ACh-binding subunit, α_γ_, drives the conformational change by pushing against the extracellular part of the β subunit (wide arrow), causing the membrane part of this subunit to tilt outwards (thin arrow). To achieve the required outward tilt, and hence productive coupling between the extracellular and membrane parts, the δ subunit must resist the force transmitted through β that would push against it. We suggest that the cholesterol-rich microdomain helps to impose this resistance by reducing the free volume available for molecular motion in the hydrophobic portion of the bilayer (Song *et al.*, 2014 ▸), hence conferring rigidity next to δ. Without a supporting wall of rigid sterol groups, the required shift-to-tilt coupling of the β subunit might not be favoured, leading to an unproductive conformational change when ACh binds to the receptor, rather than conversion to an open channel.

To corroborate this suggestion and illuminate further how the lipids assist in channel opening, more detailed structural information on the lipids in the intact membrane will be required. This should be achievable by the helical reconstruction approach used in the present study when applied to more images of tubes. Of particular interest are the organization and extent of mobility of the microdomain lipids. Since they span both channels of the dimer completely, it seems possible that in addition to their anchoring effect (Fig. 4 ▸) they are able to mediate cooperative interactions between the paired channels, thereby accounting for the synchronous gating activity shown by single-channel conductance experiments (Schindler *et al.*, 1984 ▸).

## 5. Conclusion   

This paper extends a time-resolved cryo-EM study of gating of membrane-embedded ACh-receptor channels by examining the distribution of lipids surrounding the channels in the outer leaflet of the bilayer, where the displacements are greatest. The results suggest that cholesterol, in addition to stabilizing the protein, may be needed to achieve local rigidity of the membrane so that a productive conformational change takes place when ACh binds, triggering the channel to open.

## 6. Related literature   

The following reference is cited in the Supporting Information for this article: Wennberg *et al.* (2012 ▸).

## Supplementary Material

Supplementary Figures S1 and S2.. DOI: 10.1107/S2052252517005243/hi5645sup1.pdf
