# Supplementary material for: Segregation of lipids near acetylcholine-receptor channels imaged by cryo-EM
Source: IUCrJ. 2017 May 2;4(Pt 4):393–9. doi: 10.1107/S2052252517005243 (PMC5571802; doi:10.1107/S2052252517005243)
Supplement: Supplementary file 1 [file m-04-00393-sup1.pdf]

IUCrJ

Volume 4 (2017)

Supporting information for article:

Segregation of lipids near acetylcholine-receptor channels imaged by cryo-EM

Nigel Unwin

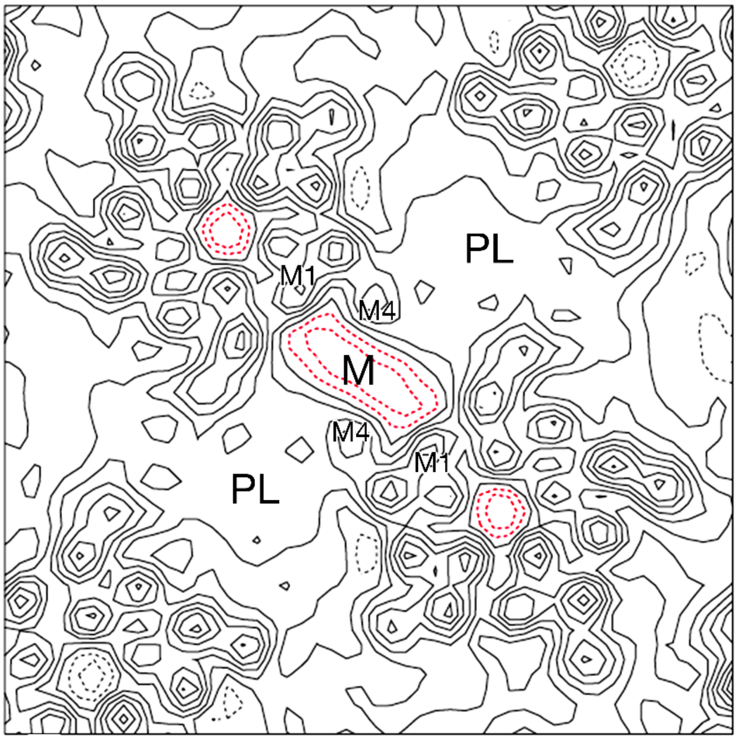

Figure S1 Contour map of the densities in the middle of the outer headgroup region, corresponding to the boxed area in Fig. 3. The phospholipid headgroups (PL) give rise to a fairly uniform positive density in the space between individual protein molecules, because of their (presumably) random disposition. The space framed by the M1 and M4 helices of the δ subunits, and associated with the microdomain (M), is an exception. The weaker density here is equal to that of the lumen of the pore, indicating a depletion of the large phospholipid headgroups and their substitution by water. The presence of cholesterol in the underlying membrane explains this anomaly, since cholesterol exposes only a hydroxyl and contributes no mass this far from the hydrophobic core of the membrane (for examples of simulated cholesterol-containing membranes, see: http://cmb.bio.uni-goettingen.de/cholmembranes.html). Contours are at 0.6σ, with the relevant microdomain and pore regions in red.

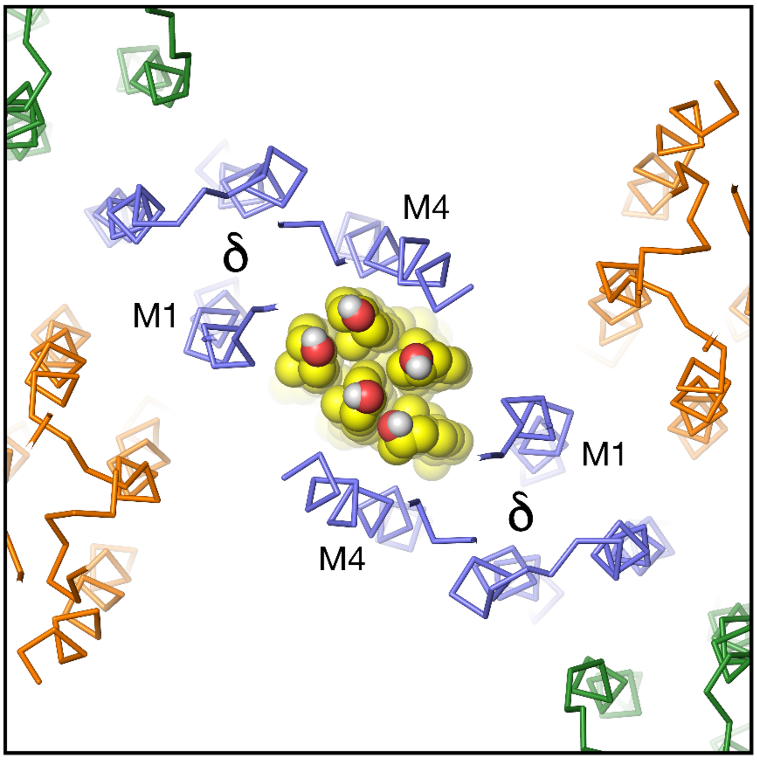

Figure S2 Hypothetical depiction of a cluster of five cholesterol molecules located in the microdomain space between the δ subunits of neighbouring receptors. The figure suggests that the microdomain, fully occupied, could harbour as many as eight cholesterols. The co-ordinates for the cluster were taken from a molecular dynamics simulation of a DOPC bilayer containing 30 mol % cholesterol (Wennberg *et al.*, 2012). The co-ordinates for the receptors were obtained by fitting the atomic model (PDB entry 2bg9) to the densities corresponding to a dimer in the 3D map; subunit colours as in Fig. 2 (α_δ, orange; β, green; δ, blue).
